# Fluorescence Visualization of the Enteric Nervous Network in a Chemically Induced Aganglionosis Model

**DOI:** 10.1371/journal.pone.0150579

**Published:** 2016-03-04

**Authors:** Takumi Fujimura, Shinsuke Shibata, Naoki Shimojima, Yasuhide Morikawa, Hideyuki Okano, Tatsuo Kuroda

**Affiliations:** 1 Department of Pediatric Surgery, Keio University School of Medicine, Shinjuku Tokyo, Japan; 2 Department of Physiology, Keio University School of Medicine, Shinjuku, Tokyo, Japan; 3 Department of Pediatric Surgery, International University of Health and Welfare, Ohtawara, Tochigi, Japan; Temple University School of Medicine, UNITED STATES

## Abstract

Gastrointestinal motility disorders, severe variants in particular, remain a therapeutic challenge in pediatric surgery. Absence of enteric ganglion cells that originate from neural crest cells is a major cause of dysmotility. However, the limitations of currently available animal models of dysmotility continue to impede the development of new therapeutics. Indeed, the short lifespan and/or poor penetrance of existing genetic models of dysmotility prohibit the functional evaluation of promising approaches, such as stem cell replacement strategy. Here, we induced an aganglionosis model using topical benzalkonium chloride in a P0-Cre/GFP transgenic mouse in which the neural crest lineage is labeled by green fluorescence. Pathological abnormalities and functional changes in the gastrointestinal tract were evaluated 2–8 weeks after chemical injury. Laparotomy combined with fluorescence microscopy allowed direct visualization of the enteric neural network *in vivo*. Immunohistochemical evaluation further confirmed the irreversible disappearance of ganglion cells, glial cells, and interstitial cell of Cajal. Remaining stool weight and bead expulsion time in particular supported the pathophysiological relevance of this chemically-induced model of aganglionosis. Interestingly, we show that chemical ablation of enteric ganglion cells is associated with a long lifespan. By combining genetic labeling of neural crest derivatives and chemical ablation of enteric ganglion cells, we developed a newly customized model of aganglionosis. Our results indicate that this aganglionosis model exhibits decreased gastrointestinal motility and shows sufficient survival for functional evaluation. This model may prove useful for the development of future therapies against motility disorders.

## Introduction

Gastrointestinal motility disorders are due to congenital enteric nervous system abnormality, inflammation and endocrine disorders. Congenital disorders are representative in the field of pediatric surgery. Hirschsprung disease (HD), a congenital gastrointestinal (GI) tract disorder, causes severe constipation due to colonic dysmotility. Absence of enteric ganglion cells originating from neural crest cells (NCCs) is its main feature, and is caused by arrest of the craniocaudal migration of NCCs during development [1–5]. The enteric nervous system (ENS) is derived from vagal and sacral neural crests, and innervates into the GI tract. Cells of the ENS undergo extensive migration, proliferation, differentiation, and survival [6].

Newborn patients suffering from HD have no or limited numbers of ganglion cells in a part of or the entire large intestine or in antecedent parts of the GI tract. The symptoms of HD include failure of meconium evacuation and constipation. The standard therapy for HD is a surgical operation, referred to as pull-through procedure. Even after this surgery, some HD patients continue to suffer from constipation or insufficient evacuation [7]. There is no definitively reparative procedure for HD patients whose aganglionic lesion extends to the stomach or esophagus. Strategies for the development of novel therapeutic approaches are thus needed if a fundamental cure is to be achieved.

Our research group focuses on cell transplantation as a new candidate therapeutic approach in HD. However, the limited range of mouse models remains a significant problem in HD research. The following features are important for any model intended for use in the evaluation of the effects of cell transplantation; 1) reliable deletion of ganglion cells; 2) consistent symptoms of dysmotility; 3) sufficient length of survival; 4) simple procedure for model generation. Several genetically induced dysmotility models with aganglionic GI tracts have been reported; however, all have limitations, in that the short lifespans of the resultant animals prevent the evaluation of long-term functional bowel recovery.

More recently, the *Ret*^9/-^ mouse strain was generated, and this model overcomes the problem of reduced life span [8]. It also offers the advantage of allowing the visualization of the ENS network through the fluorescent protein Cyan knocked into the *Ret* gene. Although this strain closely mimics the natural history of HD, the limited penetrance and length of aganglionic segment make it difficult to evaluate cell transplantation efficiency. Model mice with an ENS-specific ablation and a long life span, designated as a form of chemically-induced HD model or BAC model [9, 10] have been described previously. The ganglion cells of the colon are reliably and selectively ablated, mimicking a clinically important symptom of HD [11]. In this study, we used a chemically-induced model to generate aganglionosis, seeking to take advantage of its irreversibility and reproducibility.

In order to visualize the aganglionic segment in live animals using this model, we applied BAC treatment to a transgenic line in which the ENS is labeled with green fluorescent protein (GFP) (P0-Cre/GFP mouse) [12–15]. Here we established a chemically-induced aganglionosis mouse model with a fluorescence-labeled enteric neural network. This model enables us to observe the aganglionic segment in the living state, and to evaluate pathological and functional abnormalities over long periods.

## Materials and Methods

### Animals

A transgenic mouse expressing Cre-recombinase under the control of protein zero (P0) promoter (P0-Cre) were mated with EGFP reporter transgenic mouse (CAG-CAT-EGFP) to obtain a P0-Cre/CAG-CAT-EGFP (P0-Cre/GFP) double-transgenic strain, in which neural crest derivatives are labeled with GFP within various tissues, including the ganglion cells of the GI tract [12–17]. This animal experiment (approval number: 09125, issued date: August 1. 2012) was fully approved by the Keio University Institutional Animal Care and Use Committee in accordance with the Institutional Guidelines on Animal Experimentation at Keio University.

### Chemically-Induced Aganglionosis Model

Male P0-Cre/GFP mice (four weeks old, *n* = 75) were deeply anesthetized using isoflurane (Abbott co, Japan). Laparotomy with a midline skin incision and a preparation of small window (about 5 mm diameter) were prepared on the avascular area of the mesentery. Approximately 10 mm of the sigmoid colon, approximately 3 cm from the anal verge, was wrapped with a piece of filter paper (10 × 15 mm) soaked in 0.1% benzalkonium chloride (BAC, NIHON PHARMACEUTICAL CO, Japan) in saline for 15 min. An additional 100 μl of 0.1% BAC solution was added every 5 min on the filter paper to counter dry-up. After removal of the filter paper, the chemically-treated colon and abdominal cavity were thoroughly washed with 10 ml of sterile saline. At the end of surgery, the wound was stitched in layers. The peritoneum and skin were closed with interrupted sutures using a 6–0 nylon thread. The mortality rate after BAC treatment was 12.0% (9 deaths out of 75 model mice).

### Live Visualization of Ablated Enteric Plexus by fluorescence

Model mouse was deeply anesthetized using isoflurane (Abbott co, Japan) and performed laparotomy with a midline skin incision. The aganglionic segment was confirmed during and immediately after this operation, and seven days after BAC treatment under a fluorescence microscope (SVX10, Olympus) as GFP defect area. To confirm the effect of BAC treatment, the same individual model mouse was deeply anesthetized and examined by laparotomy several times.

### Histological Evaluation with Immunostaining

Histological analyses were performed as described [18]. In brief, deeply anesthetized mice were perfused with 0.1 M PBS and 4% PFA in 0.1 M PBS, pH 7.4 and post-fixed with the same fixative. The mucosal epithelium was removed and an enteric plexus was exposed. Direct fluorescence without immunostaining was examined under a fluorescence microscope (SVX10, Olympus, BZ-9000, and Keyence). The specimens were incubated with primary antibodies for 12 hours at 4°C, followed by incubation with secondary antibodies for one hour at room temperature. Secondary antibodies conjugated with Alexa 488, Alexa 555, or Alexa 647 (Invitrogen) were used to recognize specific primary antibodies along with nuclear staining (10 μg ml –^1^, Hoechst 33258, Sigma). The stained samples were observed with a confocal laser scanning microscope (LSM700, Carl Zeiss).

The primary antibodies used in this study were rabbit polyclonal PGP 9.5 (1:1000, Ultra Clone), rat monoclonal anti-GFAP (1:500, Invitrogen), rabbit polyclonal anti-GFP (1:500, MBL), goat polyclonal anti-GFP (1:250, Rockland), anti-CD31(1:500, BD), anti-αSMA (1:500, Sigma), AIC (anti-ICC antibody, 1:250, Cosmo Bio), rabbit polyclonal anti-CGRP (1:1000, BioMol), rabbit polyclonal anti-SubP (1:1000, Immunostar), rabbit polyclonal anti-VAChT (1:1000, Sigma), sheep polyclonal anti-TH (1:2000, Millipore). Alexa 488/555/647 conjugated donkey anti-goat IgG, donkey anti-mouse IgG, donkey anti-rabbit IgG, and goat anti-rat IgG (1:1000, Invitrogen).

### Apoptosis Detection

The ApopTag kit (Merck Millipore co.), which detects single- and double-strand breaks associated with apoptosis (TUNEL assay) was used to evaluate apoptotic activity. Specimens were incubated with equilibration buffer for 10 sec at room temperature, and incubated with TdT enzyme for 1 h at 37°C with labeling by digoxigenin-dNTP. By stopping the reaction with stop solution for 10 min followed by washing 3 × with 0.1 M PBS, samples were stained with rhodamine conjugated anti-digoxigenin antibody for 30 min and washed 4 × with 0.1 M PBS at room temperature.

### Functional Analysis of the GI motility

To functionally evaluate GI motility and constipation, we monitored remaining stool weight, bead expulsion time, body weight change, fecal pellets and food intake. To minimize the influence of the surgical operation, the same operation without BAC treatment was performed for the control group. Remaining stool weight (aganglionosis model *n* = 5, controls *n* = 5) was measured at eight weeks after BAC treatment. Just before the histological evaluation, approximately 50 mm lengths of fixative perfused colon was dissected and measured using a digital scale. The BAC treated area was located on the edge of the anal slide in the examined colon with remaining stool. Beads expulsion time (aganglionosis model *n* = 5, controls *n* = 5) was carried out as described previously [19, 20]. Briefly, 2 mm diameter beads were inserted in the colon using a plastic tip about 2 cm away from anus without anesthesia. Expulsion time was measured *in vivo* every 30 minutes after insertion as a measure of transition latency in the colon. Changes in body weight, fecal pellet count, and food intake in the model mice (aganglionosis model *n* = 25, controls *n* = 25) was measured for eight weeks. Animal weights of each week were measured twice using a digital scale (A&D, Japan), and the heavier was selected. The number of daily fecal pellets for each group (aganglionosis model *n* = 25, controls *n* = 25) was measured twice per week. Pellets were picked up individually, all bedding was removed and the pellets were dried at room temperature until feces were separated from each other. Daily food consumption was calculated by subtracting the measured weight of food from the amount measured the previous day (aganglionosis model *n* = 25, controls *n* = 25).

## Results

### Fluorescence Labeling of Enteric Plexus in Intact Colon of P0-Cre/GFP Mouse

**Fig 1:**
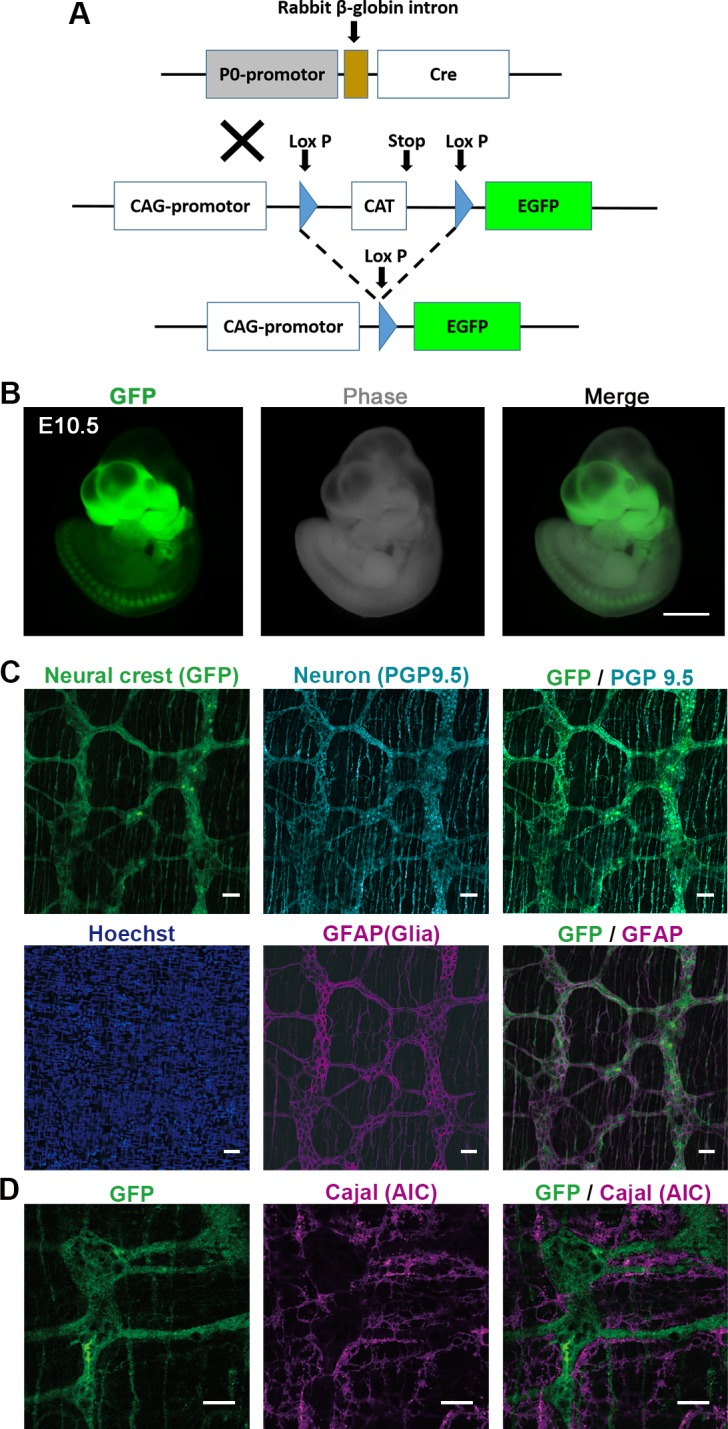
Fluorescence Labeling for Enteric Plexus in Intact Colon of P0-Cre/GFP Mouse. (A) P0-Cre/GFP mouse is a double transgenic mouse crossed between P0-Cre and CAG-CAT-EGFP transgenic strains, in which NCCs are labeled with GFP. NCCs were permanently labeled with ubiquitous promoter driven GFP induced by the transient activation of Cre-recombinase under the control of the P0 promoter. (B) At embryonic day 10.5 (E10.5), GFP positive cells are specifically localized in the craniofacial area and dorsal root ganglia (DRG) in live mouse embryo. (C) GFP-positive network structure of enteric ganglia originated from NCCs was observed in the intact colon of adult P0-Cre/GFP mouse. GFP-positive ganglion cells showed the expression of neural marker PGP 9.5 along with the GFAP positive glial cells confirmed with immunohistochemistry. (D) AIC antibody, which recognizes the interstitial cell of Cajal, was not colocalized with the GFP. Scale bars: (B) 500 μm, (C) 50 μm, (C) 20 μm

We employed the neural crest lineage-labeled mice to visualize the enteric plexus with a fluorescent protein *in vivo*. The P0-Cre/GFP double transgenic strain was obtained by intercrossing between a Cre recombinase-expressing line under the control of the P0 promoter (P0-Cre) and a GFP reporter line (CAG-CAT-EGFP) (Fig 1A) [12, 14]. In this strain, neural crest derivatives are labeled with EGFP in various tissues, including the GI tract (Fig 1B) [13]. To confirm GFP-expressing cell types in the colon in this strain, we performed immunohistochemical analysis in 4-week-old mice. It shows the GFP-positive network, comprising descendants of the neural crest, labeled with the neural marker PGP 9.5 and GFAP-positive glial cells (Fig 1C). Another important population of cells, the interstitial cell of Cajal (ICC), which contribute to pace-making in gut contraction, are recognized by the AIC antibody [21]. The AIC-positive cells did not overlap with GFP, and localized around the GFP-positive ganglion cell networks (Fig 1D). Thus, the enteric neural plexus is clearly visualized with fluorescence in the P0-Cre/GFP double transgenic mouse strain. We have carried out the immunostaining to label parasympathetic nerves with P0-Cre/GFP mouse gut, as follows. To investigate the subtype of the GFP^+^ cells in P0-Cre/GFP gut, the staining for the subtype specific markers including calcitonin gene-related peptide (CGRP), substance P (SubP), vesicular acetylcholine transport protein (VAChT) (S1A–S1C Fig) and tyrosine hydroxylase (TH) (S1D Fig). GFP expression was not detected in CGRP^+^, SP^+^, VAChT^+^ but partially detected in TH^+^ cells. These results indicate that GFP in this model mainly labels the components of the enteric nervous system that migrated from vagal neural crest and a part of extrinsic sympathetic nerve fibers.

### Live Visualization of Ablated Enteric Plexus by GFP

**Fig 2:**
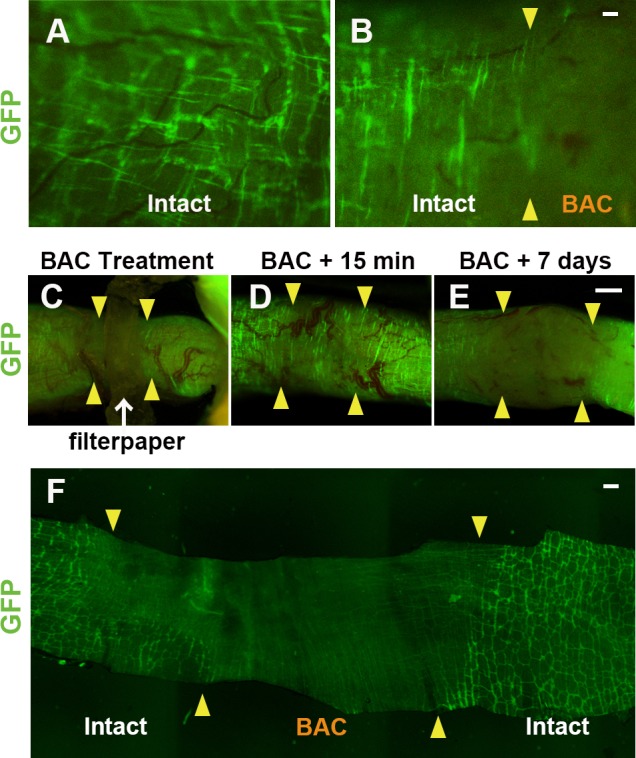
Live Visualization of Ablated Enteric Plexus by GFP. (A-E) In the live animal colon, ablation of the enteric plexus was clearly observed via uneven localization of GFP with P0-Cre/GFP mouse (A and B). In an intact colon (A), live enteric neural network consists from GFP positive ganglion cells originated from the neural crest was visualized with fluorescence without any fixation or staining. In a chemically-treated live colon (B), the junction between intact and ablated site can be detected (arrowheads in B). (C) During BAC-treatment with filter paper, the changes of the ENS network are clearly visualized with GFP. (D) Fifteen minutes after ablation, disappearance of the enteric plexus was confirmed under a fluorescence microscope. (E) Seven days after treatment, the change of the enteric ganglion network was continuously monitored by re-anesthetizing the same animal. (F) Direct GFP fluorescence clearly demonstrated the chemically-induced change of the enteric neural network just after the fixative perfusion followed by the removal of the epithelium. Arrowheads: junction between normal and ablation site, Scale: (B) 50 μm, (E), 200 μm, (F) 1 mm

To reveal alterations of the enteric neural network, we applied BAC treatment to obtain a newly customized aganglionosis model in P0-Cre/GFP mice, to enable live fluorescence imaging of the network *in vivo*. The GFP-positive network was diminished immediately after BAC treatment. Under high magnification (× 10 objective), the live intact enteric plexus can be clearly visualized with green fluorescence (Fig 2A) and the ablated part two days after BAC treatment can be easily distinguished under a fluorescence microscope (Fig 2B).

To monitor changes to the ENS after BAC treatment, the same mouse was observed following BAC treatment with repeated anesthesia. Depending on changes in the GFP^+^ network, the ablated region on the colon could be clearly observed while applying BAC (Fig 2C) and 15 minutes after treatment (Fig 2D). By re-anesthetizing the same animal, the ENS changes can be captured seven days after the operation (Fig 2E). Fluorescence visualization of the ENS *in vivo* enables us to follow up the change of GI plexus in the identical animal by using this P0-Cre/GFP strain. To evaluate the direct effects of BAC treatment on GFP, the change in fluorescence intensity was observed under a fluorescence microscope before and after BAC treatment on fixed GFP-positive cells. The comparison between the BAC-treated and -untreated control gut showed a slight decrease in GFP intensity following BAC treatment (S3 Fig). We suggest that the rapid loss of GFP in Fig 2D (15 minutes) is not mainly due to the disappearance of the GFP marker, but rather the disappearance of GFP^+^ cells. An approximately 10 mm length of aganglionic segment was constantly prepared in the chemically-induced model mice. To observe the enteric plexus condition two weeks after BAC treatment, aganglionic segment of the colon was observed without immunostaining after fixative perfusion and the wholemount tissue preparation with epithelium removed (Fig 2F). The mean length of aganglionosis in the colon of our chemically ablated models was constant between 8.2 and 16.5 mm (mean length: 12.2 ± 2.7 mm, n = 7) with 10 mm length of filter paper. The length of aganglionosis can easily be modified by the size of the filter paper in our model, which means that it is possible to control the degree of severity.

In the living animal, the ablated portion of the chemically-induced aganglionosis model was clearly visualized *in vivo*.

### Effects of Chemical-Treatment Confirmed by Immunostaining

**Fig 3:**
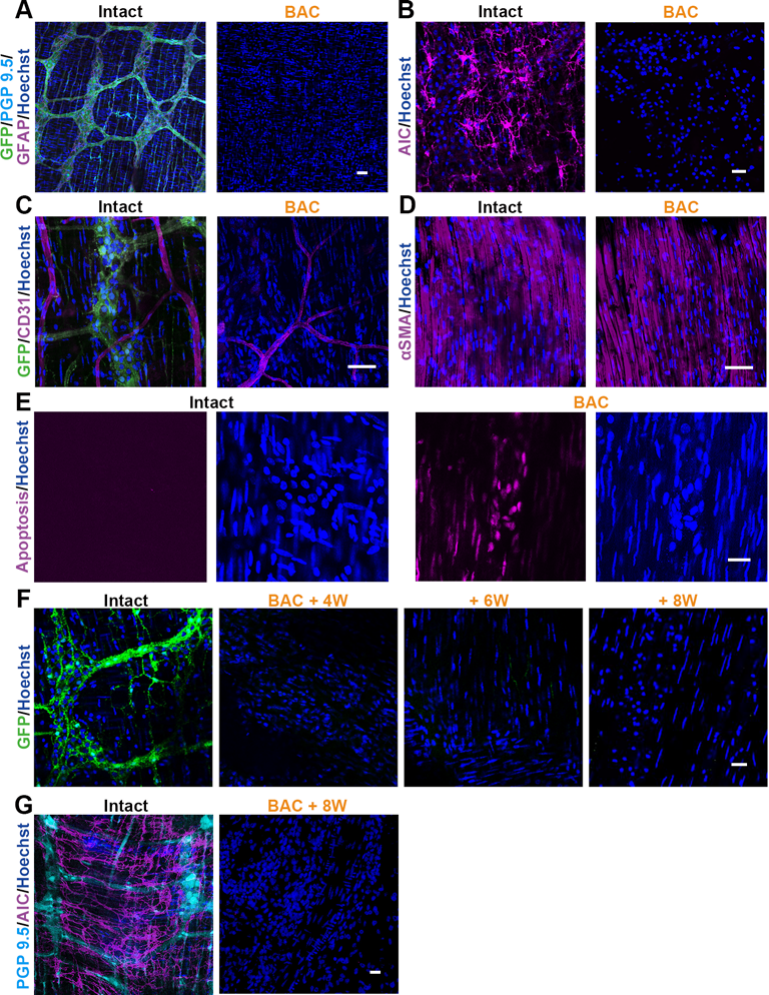
The Effects of BAC Treatment Confirmed by Immunostaining. Two weeks after BAC treatment, immunohistochemically evaluated the GFP-positive NCCs and various markers in the lesion sites. (A-D) NCCs stained with GFP antibody was entirely deleted from the BAC treated colon (A). Not only PGP 9.5-positive enteric ganglion cells, but also GFAP-positive glial cells were disappeared (A). Additionally, pacemaker cells, known as interstitial cells of Cajal (B), were also absent from the lesion sites. (C, D) No obvious defects were observed with the CD31-positive endothelial cells (C) and the αSMA-positive smooth muscle cells (D) in the ablated area. (E) Not in the intact colon, but in the BAC treated colon, the apoptosis marker was frequently detected by the ApoTag kit one day after BAC treatment. (F) Time course dependent change of the ablated segment was demonstrated that the enteric neural networks were irreversibly absent for 8 weeks after the BAC application. (G) At this time point (8 weeks), ICC was also irreversibly absent, similar to the case in the ENS networks. Scale: (A-D, F) 50 μm, (E, G) 20 μm

In order to identify the cell type-specific effect of BAC treatment in the colon, immunohistochemistry was carried out using anti-GFP antibody and various cell type-specific markers. In wholemount tissue preparations from P0-Cre/GFP colon two weeks after ablation, the GFP-positive neural network co-labeled with PGP 9.5 along with the GFAP-positive glial network were completely ablated (Fig 3A). In addition to the enteric plexus, AIC-positive ICC was also decreased in the treated area (Fig 3B). Vascular endothelial cells and smooth muscle cells showed almost no damage in chemically treated colons (Fig 3C and 3D).

To confirm whether the cells survived or not in the ablated area, apoptosis detection was carried out for 24 hours after BAC application. Only the chemically treated group demonstrated a large number of apoptotic cells in the layer of the neural plexus in the colon (Fig 3E). To identify the cell type specificity of the apoptotic cells, we immunostained the ENS 12 hours after chemical treatment. By the time point, cells highly positive for an apoptosis marker had already lost the expression of neuronal marker, and slightly positive cells were also labeled with a smooth muscle marker (S2 Fig). These results suggest that the ganglion cells died by apoptosis, and that a limited number of smooth muscle cells was also damaged by chemical treatment. The alteration of ablated GFP^+^ cells was irreversibly observed for 2–8 weeks after surgery (Fig 3F and S4 Fig). In addition, the ICC was also irreversibly ablated eight weeks after BAC treatment, which we confirmed by immunostaining with AIC antibody, as in the neural networks, above (Fig 3G).

Neural cells, glial cells and ICC were completely ablated by apoptosis after chemical treatment, but most of the endothelial cells and smooth muscle cells were survived.

### Quantitative Evaluation for Functional Alteration of GI Tract

**Fig 4:**
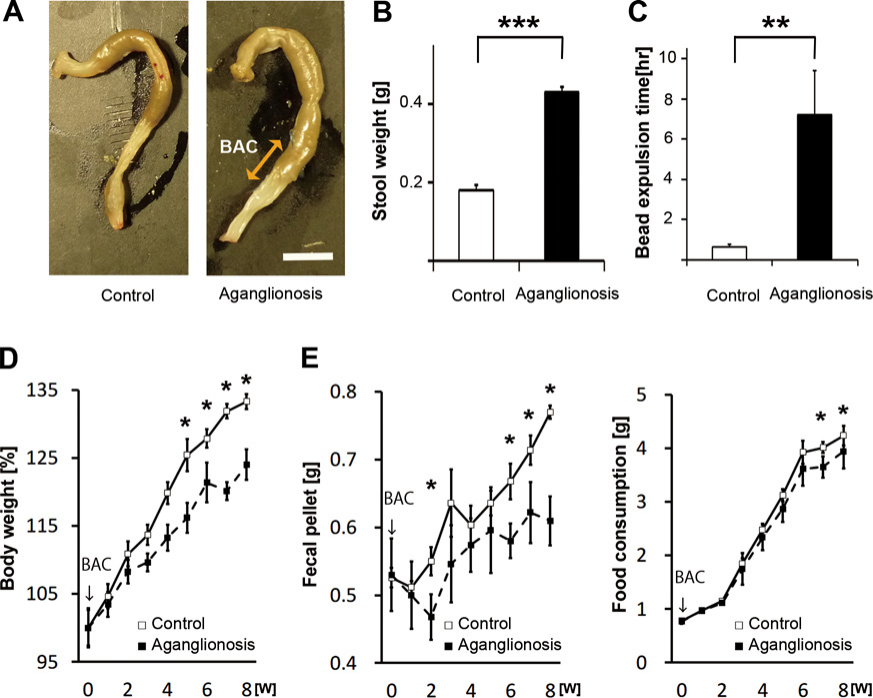
Functional Alteration of the GI Tract Was Quantitatively Evaluated. Significant functional defects from the dysmotility of the colon were observed over time after the surgery. Remaining stool weight, beads expulsion time, body weight, fecal pellet count and food consumption scaling was carried out in the chemically-treated group, with the sham operated group as a control. (A, B) Appearance of the dilated and constipated colon was demonstrated in (A). The stool weight remaining in the chemically treated mice colon (*n* = 5) was markedly heavier than that in control (*n* = 5). (C) The bead expulsion time was measured for evaluating the transition latency of the colon. The excretion period of the beads from the anus in the aganglionosis model (*n* = 5) was significantly delayed compared to control (*n* = 5). (D) Body weight gain was evaluated for eight weeks after the operation, and the ratio was significantly smaller in BAC group (*n* = 25) than that in control (*n* = 25). Values are calculated on the basis of the weight from the date of surgery (4-week-old). (E, F) Fecal pellet count and food consumption were measured for eight weeks after the operation. Significantly reduced fecal pellet count and food consumption were observed in the BAC group (*n* = 25) than in the control group (*n* = 25). Double-headed arrow: BAC treated region, Arrows: time point of BAC treatment, * *p* < 0.05, ** *p* < 0.01, *** *p* < 0.0001, Error bars: standard error.

To investigate the GI tract function of this aganglionosis model, the remaining stool weight, bead expulsion time, body weight change, fecal pellet count and food consumption were evaluated. Eight weeks after treatment, the aganglionosis model had a narrow segment and dilated colon at the proximal side of the chemically treated area (Fig 4A). Stool retention was often observed in the area proximal to the treated segment. The remaining stool weight of the aganglionosis model (*n* = 5) was significantly higher than that of the control group (*n* = 5) (*p* < 0.001, in Fig 4B). Bead expulsion time from the colon was also measured to evaluate the disturbance of defecation. The transient time of bead from the colon of the aganglionosis was significantly delayed compared to that of the control mice (*p* < 0.01, in Fig 4C). The ratio of body weight gain in the aganglionosis model (*n* = 25) was significantly smaller than that in the control (*n* = 25) from five weeks after chemical-treatment or later (*p* < 0.05, in Fig 4D). The weekly fecal pellet count of the aganglionosis model was significantly smaller than that in the control group (*n* = 25) from six weeks after BAC treatment or later (*p* < 0.05, in Fig 4E). Significant reduction of the weekly food consumption in the model (*n* = 25) was also detected compared to the control group at seven weeks after surgery (*n* = 25) (*p* < 0.05, in Fig 4F).

All these results indicate that functional defects in the aganglionosis model were quantitatively confirmed following treatment.

### Conclusions

We developed a customized aganglionosis model using P0-Cre/EGFP mouse, in which neural crest lineage cells are labeled with GFP. This model enabled us to observe the live ENS network with GFP-derived fluorescence and to evaluate functional defects of the GI tract. This model may be useful for exploring the novel regenerative therapies for aganglionic disorders of the GI tract.

## Discussion

To explore the new therapy for the patients suffering from GI motility disorder, it is quite essential to develop a useful aganglionosis model optimized for research. We employed the NCC lineage tracking P0-Cre/GFP mouse to generate a chemically induced aganglionosis mouse (Figs 1 and 2). This model of aganglionosis model offers a number of advantages over other currently available models. First, aganglionic segments are recognized by the disappearance of fluorescence during the surgical operation immediately after the removal of filter paper with BAC (Fig 2A–2C). Second, the simple procedure makes it relatively straightforward to consistently generate an experimentally useful aganglionosis model (Figs 2 and 3). Third, functional alteration of the GI tract can be evaluated reproducibly thanks to the longer survival period (Fig 4).

Recent progress of the stem cells research suggested the novel therapeutic approaches against various incurable diseases such as gastrointestinal motility disorder. The stem cell therapy for motility disorders tried to apply not only for the gastroparesis, achalasia but also for the HD [4, 6, 22]. Previous studies reported that stem cells of central nervous system transplanted into the stomach of adult mice [22]. Some researchers showed that neural crest stem cells isolated from embryonic mice gut formed ENS like clusters containing neuron and glial marker positive cells in wild type mice [23, 24]. The challenges with the stem cell transplantation therapies against GI motility disorders are promising candidate by replacing or supplementing the neurons for the aberrant ENS network.

The biggest advantage of this model is the ability to visualize the ENS *in vivo*. This enables us to confirm whether we succeeded in making the chemically-induced aganglionosis model or not before the surgical suture by detecting the disappearance of the fluorescent from ENS, which can lower experimental failure rates and reduce unnecessary sacrifice of animals. This advantage also supports the chance of enhancing the precise cell transplantation owing to the clear identification of aganglionic segment under the fluorescence microscope. While screening for investigating some effective agents to ENS regeneration, we can quantitatively detect the effectiveness with living animal over the time. Additionally, it might be able to observe cell-cell interaction between the transplanted cells and original ENS network by using multiple fluorescence color labeling.

This model is easy to generate using simple techniques. Approximately 10 mm of the sigmoid colon, approximately 30 mm from the anal verge, is wrapped with a piece of filter paper soaked in 0.1% BAC in saline for 15 min. We suggest that this simple procedure will readily be applied to other genetically modified mouse strains, and that the aganglionic phenotype can be modified by changing the length of the chemically treated segment. With respect to the chemical treatment, one previous study reported that the minimum required treated segment length is about 5 mm, to prevent spontaneous recovery [25]. To obtain a sufficient length of the complete aganglionic portion in the colon, we chose to apply BAC to a 10 mm segment. Immunohistochemistry analysis for the center of the aganglionic segment eight weeks after surgery found no evident spontaneous regeneration, consistent with previous reports [10, 25]. When a 20 mm aganglionic segment in the colon was produced, the mice frequently died (over 80%) within two weeks, due to severe constipation and colon perforation. These results resembled those from a previous report of mortality rates influenced by the length of the aganglionic segment in another motility disorder model.

Previously, several motility disorder genetically induced HD models with aganglionic GI tracts have been reported; however, all have limitations, in that the short lifespans of the resultant animals prevent the evaluation of long-term functional bowel recovery. Mice of the *Ret-* and *glial cell line-derived neurotrophic factor (GDNF)*-deficient strains die soon after birth due to kidney dysplasia secondary to abnormalities in the RET-GDNF family receptor alpha 1 (GFRα1)/GDNF pathway [26]. The piebald-lethal mouse, carrying a mutation in the endothelin-B receptor, develops megacolon with a long aganglionic segment and dies within several weeks after birth due to severe constipation [27, 28]. The mean length of aganglionic colon of our BAC model was similar to some genetically induced HD model, for example, piebald lethal mice with a long lifespan. The length of our model was pretty consistent between 8.2 and 16.5 mm (mean length: 12.2 ± 2.7 mm). The length of aganglionosis in homozygous piebald lethal mice was between 12.3 and 27 mm (mean length: 20.4 ± 2.1 mm) and that in heterozygous mice was between 8.3 and 16.1 mm (mean length: 12.4 ± 1.1 mm) [27]. On the other hand, the study with other model with longer life span, Ret9/-, has shorter aganglionic segment less than 3 mm in moderate cases [8]. As for the continuity from the anus, most genetically induced HD models have continuous aganglionic segment. By contrast, drug induced model has arbitrarily-distributed aganglionosis region anywhere in the colon with arbitrarily-sized. Some researchers have also shown that if the aganglionic colon was created within the suitable length, chemically treated mice can survive 3–5 months or more [29, 30]. More recently, the *Ret*^9/-^ mouse strain was generated, and this model overcomes the problem of reduced life span. It also offers the advantage of allowing the visualization of the ENS network through the fluorescent protein Cyan knocked into the *Ret* gene. The phenotype in this model was observed with a penetrance of about 46% and over aganglionic segments of inconsistent length. The limited penetrance and length of aganglionic segment might make it difficult to evaluate cell transplantation efficiency. It was previously reported that a chemically-induced aganglionosis model mice have a longer life span due to an ENS-specific ablation [9, 10]. The reported mechanism of action for this approach is that a BAC acts as a cationic surfactant that attaches to the cell membrane, showing irreversible depolarization and causing cell membrane injury [10]. As a result, the ganglionic cells of the colon are reliably and selectively ablated, mimicking a clinically important symptom of motility disorder of GI tract [11].

This model also poses a number of limitations. First, chemically induced aganglionosis is not a mimic of HD pathogenesis, because the cause of HD is not injury. In the typical HD, absence of ENS is not capable of regeneration and ICC preserved. Second, certain invasive operations are required to investigate the *in vivo* ENS network, such as anesthesia and laparotomy for accessing the GI tract with the same animal continuously.

Compare to the HD model, previous study reported that the ENS in this model is selectively ablated; however the present study demonstrated another cell types are also damaged. Not only neural lineage cells, but also the glial cells, ICC, and smooth muscle cells were affected in the chemically-treated GI tract by immunohistochemical evaluation (Fig 3 and S2 Fig). Previously, ones described that they observed regeneration of nerve fibers in the edge of chemically-treated area and the others found that glial cells migrate into the chemically-treated region [30]. In this study, our observation focused on the center area of ablation to escape from the contamination of the spontaneous recovery (Fig 3F and 3G). Our data demonstrated in this study confirmed that this chemically-induced model mouse have certain length of complete aganglionic portion for several month. This model showed there was no obvious change with vascular system and smooth muscle in the immunostaining (Fig 3C and 3D). Our apoptosis marker study showed that there was strong expression of apoptosis marker in an area where neural marker was previously positive between the smooth muscle layers (S2 Fig). It seemed that limited number of smooth muscle cells showed the slight positive for apoptosis. These results strongly suggested that BAC treatment affected not only on neuron but also on other types of cells including glial cells, ICC and smooth muscles. Neuron and glial cells seemed to be more easily damaged than the smooth muscle by chemical treatment (Fig 3A–3D). Limited number of human patients suffering from repeated enterocolitis and from the enterocolitis with HD often showed the loss of ICC due to chronic inflammation of the intestine [31, 32]. Thus, the phenotypes of our chemically induced model show a slight overlap with the clinical symptoms.

We performed several functional evaluations. The remaining stool weight in the colon (Fig 4A and 4B) and bead expulsion time (Fig 4C) indicated a significant change after constipation. When we sacrifice this model for obtaining specimens with the histological evaluation, we noticed that massive stools were frequently remained in the colon in this model. It’s really true that the remaining stool scaling in the colon is not a typical functional analysis; stool weight change is quite consistent and easy to carry out. The limitation of this evaluation was that there were big difficulties for detecting the time course dependent change of the remaining stool weight. We also carried out the well-known functional analysis including bead expulsion time [19, 20], body weight increasing rate, fecal pellet count and food consumption. Each analyses demonstrated a certain differences, however, remaining stool weight and bead expulsion time were the appropriate analysis especially for this aganglionosis model because of the obvious differences in the early period of the experiment.

In summary, we have developed a useful aganglionosis model using P0-Cre/GFP mouse, in which neural crest lineage cells are labeled with GFP. This model enabled us to observe the live ENS network with GFP-derived fluorescence and to evaluate functional defects of the GI tract, and may be useful for exploring the effects of innovative regenerative therapies for motility disorders.

## Supporting Information

S1 Fig The GFP expression in the autonomic nerve in P0-Cre/GFP mouse gut. The subtype of the GFP^+^ cells in P0-Cre/GFP mouse gut evaluated by immunohistochemistry with the specific markers for parasympathetic and sympathetic nerve fibers. (A-C) CGRP, SubP and VAChT, parasympathetic nerve markers, did not colocalize with GFP in P0-Cre/GFP gut. (D) The limited number of TH^+^ fibers colabelled with GFP, which indicated that the sympathetic nerves partially labeled with GFP. Scale bars, 20 μm
(TIF)

S2 Fig Cell type specificity of the apoptotic cells. ENS in the gut at 12 hours after chemical treatment was immunostained with the markers for ganglion cells, smooth muscle and apoptosis. The ganglion cell aggregation was highly positive for apoptosis marker, but negative for neuronal marker located between the smooth muscle layers. A limited number of smooth muscle cells (arrowheads) are weakly positive for apoptosis marker. Scale bar: 20 μm
(TIF)

S3 Fig Direct effect on GFP by the BAC application. The direct BAC effect for the fluorescence protein itself was evaluated with the fixed GFP^+^ cells with or without BAC application. The GFP intensity was slightly decreased after the BAC treatment observed with the fluorescence microscope. Scale bar, 20 μm
(TIF)

S4 Fig The ablated ganglion cells in myenteric and submucosal plexus. (A) In the intact colon, PGP9.5 positive ganglion cells and GFAP positive glial cells were localized both in the myenteric plexus and in the submucosal plexus. (B) After the chemical treatment, ganglion cells and glial cells are disappeared from the all layer at the center region of the ablated colon. Dotted white lines: edge of the smooth muscle layer, Scale bar: 50 μm
(TIF)

## References

[1] Badner JA, Sieber WK, Garver KL, Chakravarti A. A genetic study of Hirschsprung disease. Am J Hum Genet. 1990;46(3):568–80. 2309705 PMC1683643

[2] Amiel J, Lyonnet S. Hirschsprung disease, associated syndromes, and genetics: a review. J Med Genet. 2001;38(11):729–39. 11694544 10.1136/jmg.38.11.729 PMC1734759

[3] Parisi MA, Kapur RP. Genetics of Hirschsprung disease. Curr Opin Pediatr. 2000;12(6):610–7. . 11106284 10.1097/00008480-200012000-00017

[4] Passarge E. Dissecting Hirschsprung disease. Nat Genet. 2002;31(1):11–2. 10.1038/ng878 . 11953748

[5] Skinner MA. Hirschsprung's disease. Curr Probl Surg. 1996;33(5):389–460. . 8665807 10.1016/s0011-3840(96)80009-8

[6] Heanue TA, Pachnis V. Enteric nervous system development and Hirschsprung's disease: advances in genetic and stem cell studies. Nat Rev Neurosci. 2007;8(6):466–79. 10.1038/nrn2137 . 17514199

[7] Friedmacher F, Puri P. Residual aganglionosis after pull-through operation for Hirschsprung's disease: a systematic review and meta-analysis. Pediatr Surg Int. 2011;27(10):1053–7. 10.1007/s00383-011-2958-5 . 21789665

[8] Uesaka T, Nagashimada M, Yonemura S, Enomoto H. Diminished Ret expression compromises neuronal survival in the colon and causes intestinal aganglionosis in mice. J Clin Invest. 2008;118(5):1890–8. 10.1172/JCI34425 18414682 PMC2293334

[9] Sato A, Yamamoto M, Imamura K, Kashiki Y, Kunieda T, Sakata K. Pathophysiology of aganglionic colon and anorectum: an experimental study on aganglionosis produced by a new method in the rat. J Pediatr Surg. 1978;13(4):399–435. . 682089 10.1016/s0022-3468(78)80464-3

[10] Yoneda A, Shima H, Nemeth L, Oue T, Puri P. Selective chemical ablation of the enteric plexus in mice. Pediatr Surg Int. 2002;18(4):234–7. 10.1007/s003830100681 . 12021968

[11] Parr EJ, Sharkey KA. Multiple mechanisms contribute to myenteric plexus ablation induced by benzalkonium chloride in the guinea-pig ileum. Cell Tissue Res. 1997;289(2):253–64. . 9211828 10.1007/s004410050872

[12] Kawamoto S, Niwa H, Tashiro F, Sano S, Kondoh G, Takeda J, et al A novel reporter mouse strain that expresses enhanced green fluorescent protein upon Cre-mediated recombination. FEBS Lett. 2000;470(3):263–8. . 10745079 10.1016/s0014-5793(00)01338-7

[13] Nagoshi N, Shibata S, Kubota Y, Nakamura M, Nagai Y, Satoh E, et al Ontogeny and multipotency of neural crest-derived stem cells in mouse bone marrow, dorsal root ganglia, and whisker pad. Cell Stem Cell. 2008;2(4):392–403. 10.1016/j.stem.2008.03.005 . 18397758

[14] Yamauchi Y, Abe K, Mantani A, Hitoshi Y, Suzuki M, Osuzu F, et al A novel transgenic technique that allows specific marking of the neural crest cell lineage in mice. Dev Biol. 1999;212(1):191–203. 10.1006/dbio.1999.9323 . 10419695

[15] Nishikawa R, Hotta R, Shimojima N, Shibata S, Nagoshi N, Nakamura M, et al Migration and differentiation of transplanted enteric neural crest-derived cells in murine model of Hirschsprung's disease. Cytotechnology. 2015;67(4):661–70. 10.1007/s10616-014-9754-8 25230796 PMC4474987

[16] Tomita Y, Matsumura K, Wakamatsu Y, Matsuzaki Y, Shibuya I, Kawaguchi H, et al Cardiac neural crest cells contribute to the dormant multipotent stem cell in the mammalian heart. J Cell Biol. 2005;170(7):1135–46. 10.1083/jcb.200504061 16186259 PMC2171522

[17] Yoshida S, Shimmura S, Nagoshi N, Fukuda K, Matsuzaki Y, Okano H, et al Isolation of multipotent neural crest-derived stem cells from the adult mouse cornea. Stem Cells. 2006;24(12):2714–22. 10.1634/stemcells.2006-0156 . 16888282

[18] Shibata S, Yasuda A, Renault-Mihara F, Suyama S, Katoh H, Inoue T, et al Sox10-Venus mice: a new tool for real-time labeling of neural crest lineage cells and oligodendrocytes. Mol Brain. 2010;3:31 10.1186/1756-6606-3-31 21034515 PMC2989948

[19] Koslo RJ, Burks TF, Porreca F. Centrally administered bombesin affects gastrointestinal transit and colonic bead expulsion through supraspinal mechanisms. J Pharmacol Exp Ther. 1986;238(1):62–7. . 3755171

[20] Ono H, Nakamura A, Matsumoto K, Horie S, Sakaguchi G, Kanemasa T. Circular muscle contraction in the mice rectum plays a key role in morphine-induced constipation. Neurogastroenterol Motil. 2014;26(10):1396–407. 10.1111/nmo.12387 . 25041353

[21] Torihashi S, Yokoi K, Nagaya H, Aoki K, Fujimoto T. New monoclonal antibody (AIC) identifies interstitial cells of Cajal in the musculature of the mouse gastrointestinal tract. Auton Neurosci. 2004;113(1–2):16–23. 10.1016/j.autneu.2004.05.004 . 15296791

[22] Micci MA, Kahrig KM, Simmons RS, Sarna SK, Espejo-Navarro MR, Pasricha PJ. Neural stem cell transplantation in the stomach rescues gastric function in neuronal nitric oxide synthase-deficient mice. Gastroenterology. 2005;129(6):1817–24. 10.1053/j.gastro.2005.08.055 . 16344050

[23] Kulkarni M. Constructive interference in steady-state/FIESTA-C clinical applications in neuroimaging. J Med Imaging Radiat Oncol. 2011;55(2):183–90. 10.1111/j.1754-9485.2011.02249.x . 21501408

[24] Hotta R, Stamp LA, Foong JP, McConnell SN, Bergner AJ, Anderson RB, et al Transplanted progenitors generate functional enteric neurons in the postnatal colon. J Clin Invest. 2013;123(3):1182–91. 10.1172/JCI65963 23454768 PMC3582137

[25] Hanani M, Ledder O, Yutkin V, Abu-Dalu R, Huang TY, Hartig W, et al Regeneration of myenteric plexus in the mouse colon after experimental denervation with benzalkonium chloride. J Comp Neurol. 2003;462(3):315–27. 10.1002/cne.10721 . 12794735

[26] Schuchardt A, D'Agati V, Larsson-Blomberg L, Costantini F, Pachnis V. RET-deficient mice: an animal model for Hirschsprung's disease and renal agenesis. J Intern Med. 1995;238(4):327–32. . 7595168 10.1111/j.1365-2796.1995.tb01206.x

[27] Ro S, Hwang SJ, Muto M, Jewett WK, Spencer NJ. Anatomic modifications in the enteric nervous system of piebald mice and physiological consequences to colonic motor activity. Am J Physiol Gastrointest Liver Physiol. 2006;290(4):G710–8. 10.1152/ajpgi.00420.2005 . 16339294

[28] Spencer NJ, Kerrin A, Singer CA, Hennig GW, Gerthoffer WT, McDonnell O. Identification of capsaicin-sensitive rectal mechanoreceptors activated by rectal distension in mice. Neuroscience. 2008;153(2):518–34. 10.1016/j.neuroscience.2008.02.054 . 18395992 PMC4652643

[29] Joseph NM, He S, Quintana E, Kim YG, Nunez G, Morrison SJ. Enteric glia are multipotent in culture but primarily form glia in the adult rodent gut. J Clin Invest. 2011;121(9):3398–411. 10.1172/JCI58186 21865643 PMC3163971

[30] Laranjeira C, Sandgren K, Kessaris N, Richardson W, Potocnik A, Vanden Berghe P, et al Glial cells in the mouse enteric nervous system can undergo neurogenesis in response to injury. J Clin Invest. 2011;121(9):3412–24. 10.1172/JCI58200 21865647 PMC3163972

[31] Wang H, Zhang Y, Liu W, Wu R, Chen X, Gu L, et al Interstitial cells of Cajal reduce in number in recto-sigmoid Hirschsprung's disease and total colonic aganglionosis. Neurosci Lett. 2009;451(3):208–11. 10.1016/j.neulet.2009.01.015 . 19159660

[32] Zarate N, Mearin F, Wang XY, Hewlett B, Huizinga JD, Malagelada JR. Severe idiopathic gastroparesis due to neuronal and interstitial cells of Cajal degeneration: pathological findings and management. Gut. 2003;52(7):966–70. 12801952 10.1136/gut.52.7.966 PMC1773724

