# Supplementary figures and images for: Fluorescence Visualization of the Enteric Nervous Network in a Chemically Induced Aganglionosis Model
Source: PLoS One. 2016 Mar 4;11(3):e0150579. doi: 10.1371/journal.pone.0150579 (PMC4778943; doi:10.1371/journal.pone.0150579)

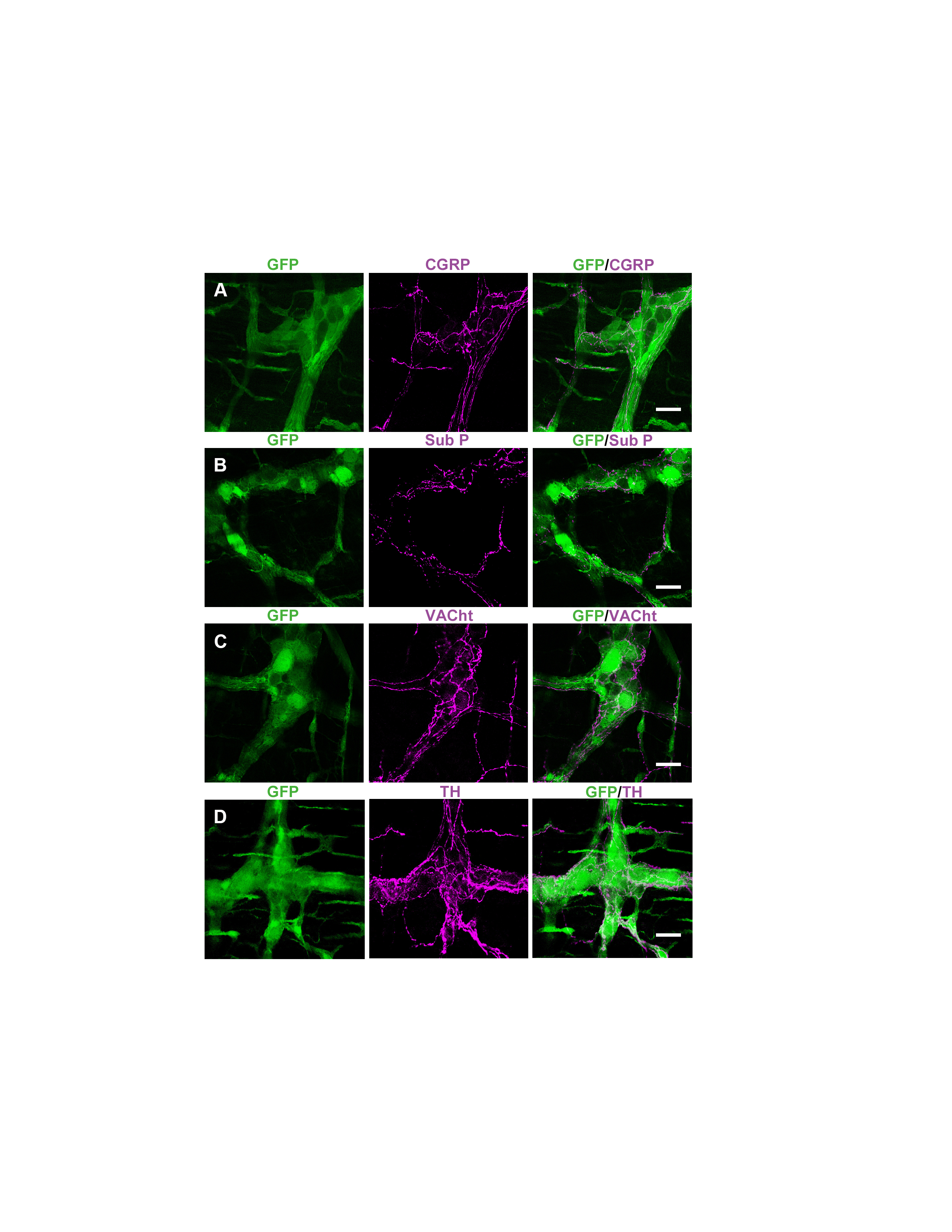

Supplement: S1 Fig — The subtype of the GFP+ cells in P0-Cre/GFP mouse gut evaluated by immunohistochemistry with the specific markers for parasympathetic and sympathetic nerve fibers. (A-C) CGRP, SubP and VAChT, parasympathetic nerve markers, did not colocalize with GFP in P0-Cre/GFP gut. (D) The limited number of TH+ fibers colabelled with GFP, which indicated that the sympathetic nerves partially labeled with GFP. Scale bars, 20 μm (TIF) [file pone.0150579.s001.tif]

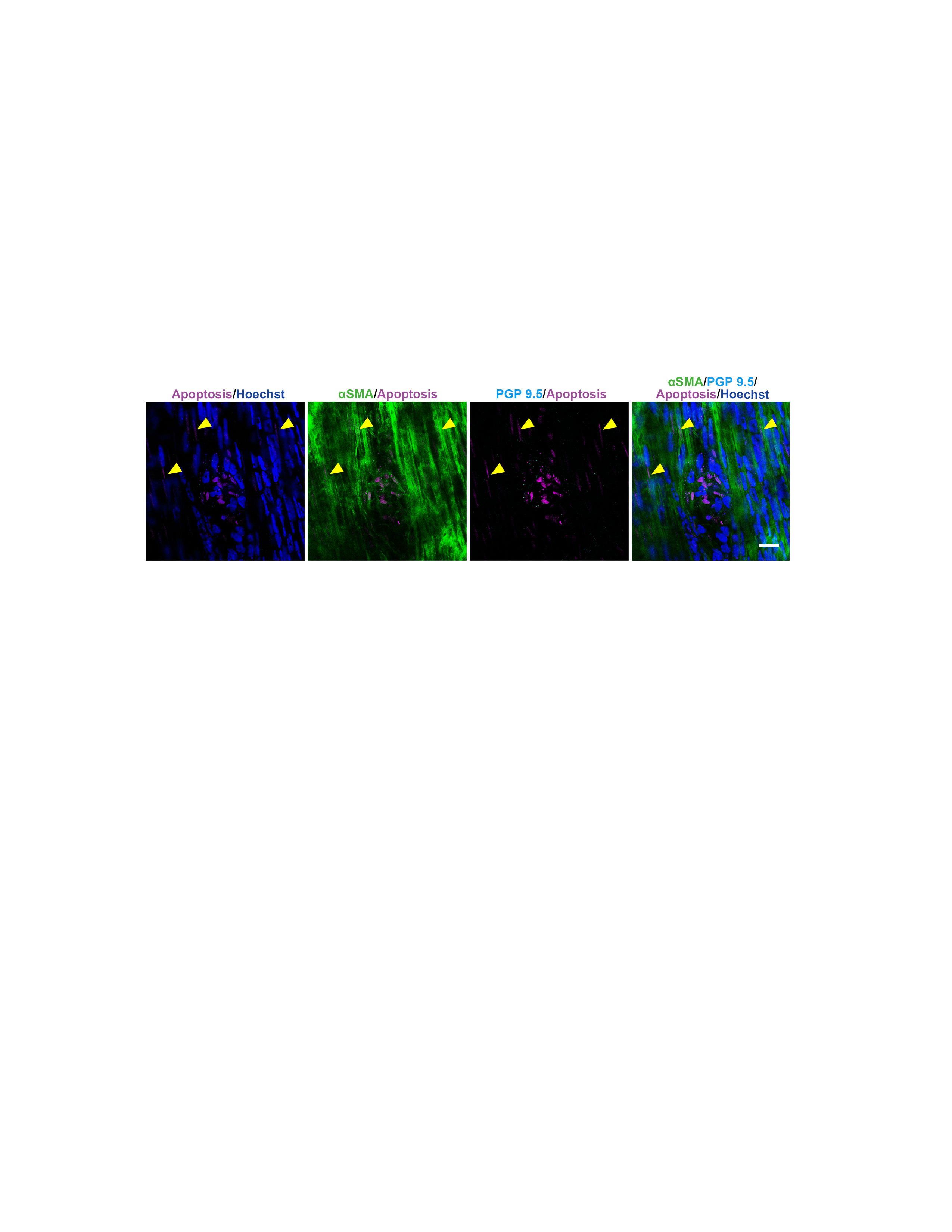

Supplement: S2 Fig — ENS in the gut at 12 hours after chemical treatment was immunostained with the markers for ganglion cells, smooth muscle and apoptosis. The ganglion cell aggregation was highly positive for apoptosis marker, but negative for neuronal marker located between the smooth muscle layers. A limited number of smooth muscle cells (arrowheads) are weakly positive for apoptosis marker. Scale bar: 20 μm (TIF) [file pone.0150579.s002.tif]

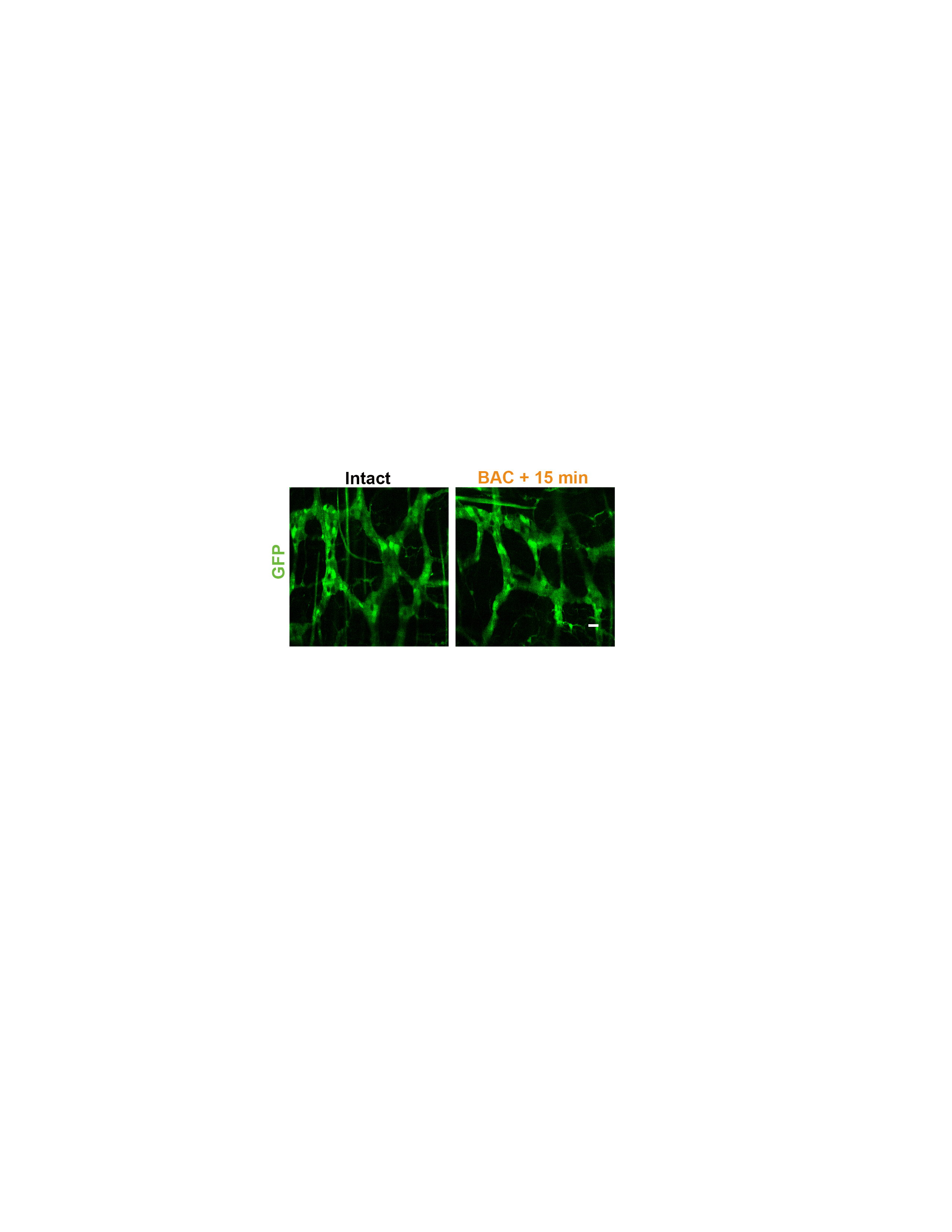

Supplement: S3 Fig — The direct BAC effect for the fluorescence protein itself was evaluated with the fixed GFP+ cells with or without BAC application. The GFP intensity was slightly decreased after the BAC treatment observed with the fluorescence microscope. Scale bar, 20 μm (TIF) [file pone.0150579.s003.tif]

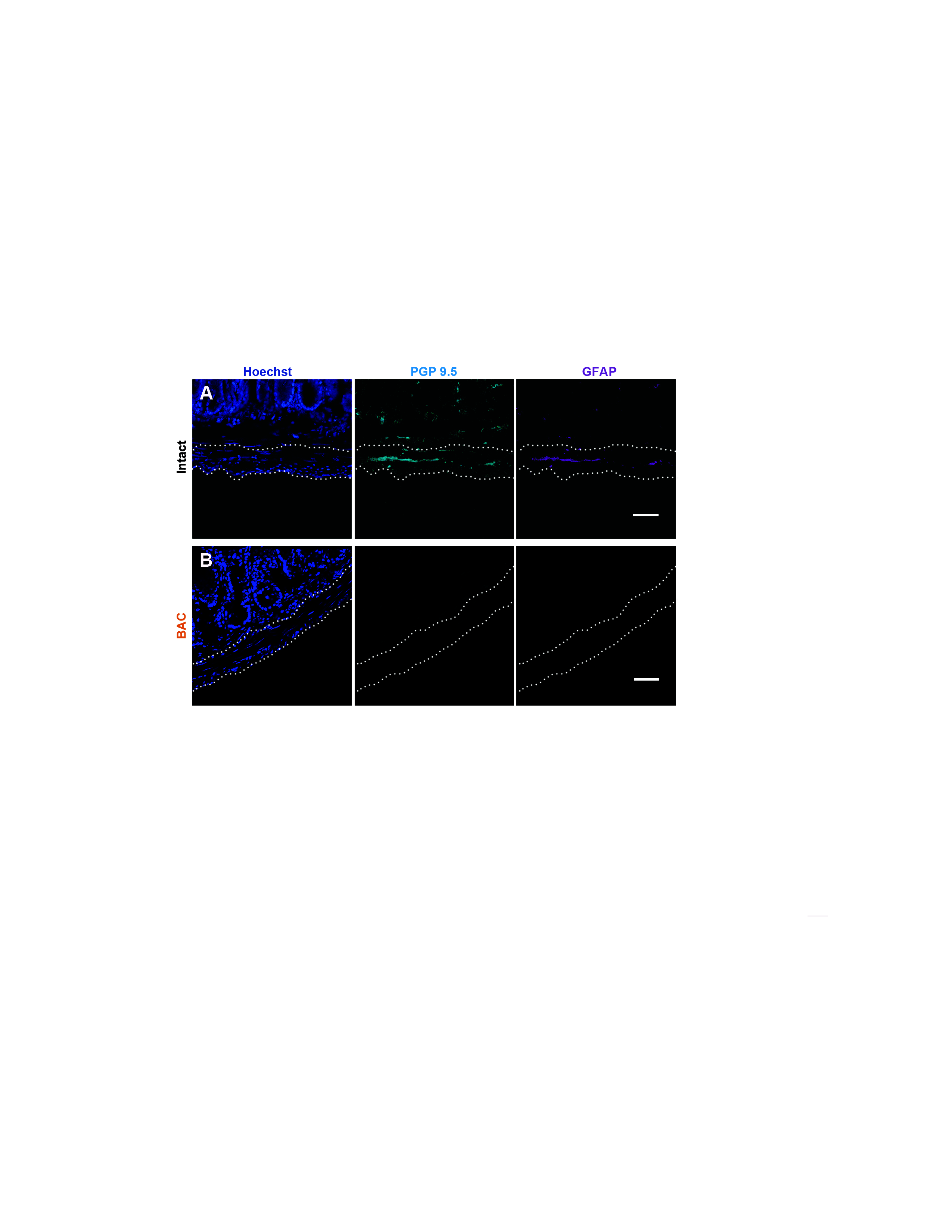

Supplement: S4 Fig — (A) In the intact colon, PGP9.5 positive ganglion cells and GFAP positive glial cells were localized both in the myenteric plexus and in the submucosal plexus. (B) After the chemical treatment, ganglion cells and glial cells are disappeared from the all layer at the center region of the ablated colon. Dotted white lines: edge of the smooth muscle layer, Scale bar: 50 μm (TIF) [file pone.0150579.s004.tif]
